# Nasal Colonization of Humans with Methicillin-Resistant *Staphylococcus aureus* (MRSA) CC398 with and without Exposure to Pigs

**DOI:** 10.1371/journal.pone.0006800

**Published:** 2009-08-27

**Authors:** Christiane Cuny, Rolf Nathaus, Franziska Layer, Birgit Strommenger, Doris Altmann, Wolfgang Witte

**Affiliations:** 1 Robert Koch Institute, Wernigerode Branc, Wernigerode, Germany; 2 Robert Koch Institute, Division of Epidemiology, Berlin, Germany; 3 Rolf Nathaus, Veterinary Practice, Reken, Germany; Columbia University, United States of America

## Abstract

**Background:**

Studies in several European countries and in North America revealed a frequent nasal colonization of livestock with MRSA CC398 and also in humans with direct professional exposure to colonized animals. The study presented here addresses the question of further transmission to non exposed humans.

**Methods:**

After selecting 47 farms with colonized pigs in different regions of Germany we sampled the nares of 113 humans working daily with pigs and of their 116 non exposed family members. The same was performed in 18 veterinarians attending pig farms and in 44 of their non exposed family members. For investigating transmission beyond families we samples the nares of 462 pupils attending a secondary school in a high density pig farming area. MRSA were detected by direct culture on selective agar. The isolates were typed by means of *spa-*sequence typing and classification of SCC*mec* elements. For attribution of *spa* sequence types to clonal lineages as defined by multi locus sequence typing we used the BURP algorithm. Antibiotic susceptibility testing was performed by microbroth dilution assay.

**Results:**

At the farms investigated 86% of humans exposed and only 4.3% of their family members were found to carry MRSA exhibiting *spa-*types corresponding to clonal complex CC398. Nasal colonization was also found in 45% of veterinarians caring for pig farms and in 9% of their non exposed family members. Multivariate analysis revealed that antibiotic usage prior to sampling beard no risk with respect to colonization. From 462 pupils only 3 were found colonized, all 3 were living on pig farms.

**Conclusion:**

These results indicate that so far the dissemination of MRSA CC398 to non exposed humans is infrequent and probably does not reach beyond familial communities.

## Introduction

Methicillin-resistant *Staphylococcus aureus* (MRSA) emerged in hospitals since the late 1960s and became a frequent nosocomial pathogen by the 1990s (health care associated, haMRSA [1]). During the past 10 years the epidemiology of MRSA changed with the appearance of infections in the community affecting people with no epidemiological connections to health care settings (community associated, caMRSA [2]). The first communication on MRSA in animals in 1972 was followed by sporadic reports on MRSA infections in various pet animal species and livestock, since the late 1990s MRSA infections in pet animal patients at veterinary hospitals became known (for summary see [3]). MRSA strains associated with these cases usually correspond to particular haMRSA strains or are related to them [3], [4]. Soon after the surprising detection of a wide nasal colonization of pigs by a particular clonal complex of MRSA, namely CC398, in the Netherlands since 2004 [5], [6], this observation has been confirmed by a number of studies in other European countries including Belgium [7] Denmark [8], Germany [9], and also in Canada [10], the USA [11] and Singapore [12]. The first report on MRSA CC398 came from a study performed in 2003 France [13].

Nearly all MRSA isolates from pigs belong to clonal complex CC398. In the mean time this clone has also been detected in other animal species such as cattle [14], chicken [15], horses [16], and also pets [9].

Several studies indicate that MRSA CC398 can be transmitted to humans as colonizer [11], [13], [17], [18], [19], and although infrequently reported so far, it is able to cause clinical infections [9], [20], [21], [22].

Above all data from the Netherlands show that a considerable number of humans with professional exposure to pigs carry MRSA CC398 [23]. For assessing the risk the wide dissemination of MRSA CC398 in intensive livestock production poses to humans at first the question of human to human transmission has to be answered. Here we report results from a study on nasal colonization of farmers and veterinarians with continuous exposure to pig farming and transmission to their nonexposed family members.

## Materials and Methods

### Selection of farms and sampling

The study was performed from September 2007 to January 2009. In a first step farms were contacted through the public veterinary sector and through contacting local veterinarians. Criteria for enrolment into the study were the farmer's work with gilts and finishing pigs as well as the consent to investigations on nasal carriage of MRSA of pigs, of the persons exposed to pigs by daily work, and of their familial human contacts. Another prerequisite was a written consent to fill a short questionnaire addressing individual factors such as age, sex, contacts with animals, previous stay in health care settings and antibiotic usage during 12 months prior to sampling. On at least 30 farms we expected at least 120 humans (on average 2 persons exposed and 2 further family members) which is sufficient to estimate MRSA point prevalence according to data on colonization of pig farmers from the Netherlands [23]. This would correspond to a 5% risk of type I error and a precision of 10% (calculation by means of Epi-Info 6.04, CDCs, Atlanta, GA, USA).

We contacted 57 farms in German Federal countries Bavaria, Lower Saxony, North-Rhine Westphalia and Saxony Anhalt, all of the farmers agreed to participate in the study. Nasal swabs were taken from pigs at different stages of fattening (15 animals per farm) and subsequently processed for detection of MRSA. Colonization of pigs was found in 47 of these farms. In case of detection of MRSA this was found for pigs at all stages of rearing. In a second step humans living on these farms with MRSA positive pigs were investigated for nasal MRSA carriage by taking swabs from both nostrils.

For studying transmission beyond families living on farms we investigated nasal swabs from pupils of a secondary school in a high density pig farming area in which 27 of the study farms are located and which is attended by children and youngsters living on these farms. To perform such kind of study in a school has the advantage of more or less close contacts between individuals in class rooms. There might be a bias because of differences in nasal colonization between children and adults as children younger 10 years are obviously more frequently colonized [24]. As the age of pupils in this school was between 10 and 16 this was probably of minor influence. Before starting sampling we obtained a written consent from the parents.

The design of the study was approved by the ethical committee of the Otto von Guericke University Magdeburg, affiliated to the faculty of medicine (file #47/09).

### Investigation of veterinarians attending pig farms

Study on veterinarians: Starting from the investigation on pig farms veterinarians caring for the 47 farms in the corresponding areas were contacted for investigation of nasal MRSA carriage of themselves and of their familial contact persons.

All of the 49 persons contacted agreed with taking nasal swabs from their own nostrils, and 15 agreed to expand this to their familial contacts.

### Microbiological analysis

Swabs were streaked on chromagar MRSA^®^ from Becton Dickinson, Heidelberg, Germany and in parallel onto Mueller-Hinton sheep blood agar (from Oxoid). After incubation at 37°C±1°C for 24 hours suspected colonies were subcultured on sheep blood agar. Confirmation of *S. aureus* was performed by demonstration of the clumping factor, by tube coagulase test using human plasma, and for all of the isolates from exposed humans and from their nonexposed family members by PCR for *tuf* [25]. For antimicrobial susceptibility testing we used microbroth dilution assay [26]. All isolates were typed by means of *spa*-typing [27]. Attribution to clonal lineages was performed by support of the BURP algorithm [28]. Isolates exhibiting new *spa*-types not attributed to clonal lineage CC398 so far were subjected to multilocus sequence typing [29].

### Assessment of transmission between humans

Demonstration of MRSA isolates exhibiting the same *spa*-type and the same antibiotic resistance phenotype in humans exposed to pigs and in their nonexposed family members was regarded as transfer. The number of transfer events within one family was calculated as the number of nonexposed MRSA positive family members, if more than one nonexposed family members was affected the number of transfer events is n - 1.

Transmission of methicillin susceptible *Staphylococcus aureus* (MSSA) was assessed by demonstration of isolates exhibiting the same *spa*-type and the same antibiotic resistance phenotype among more than one family member (number of transfer events = n−1).

#### Statistical methods

Potential risk factor associations were assessed with Fisher's exact test; univariate and multivariate analysis were performed by exact logistic regression.

## Results

### MRSA nasal colonization of humans living on pig farms

From 229 people investigated at 47 farms with MRSA colonized pigs 113 had regular daily contacts to MRSA colonized pigs, and 116 were family members living on the farm but without exposure to pigs. Results on MRSA carriage are shown in table 1. Carriage of MRSA was frequent among humans exposed to pigs (86%), with a few exceptions the *spa*-types and antibiotic resistance profiles corresponded to those of the pig isolates. Among the 116 family members who are in familial contact with humans exposed to pigs MRSA were detected in only 5 belonging to 5 different families (4.3%). These isolates exhibited the same spa-types and antibiotic resistance phenotypes as those from their exposed contact persons. As in each of the 5 families only one nonexposed family member acquired MRSA CC398 the frequency of transfer to nonexposed family members is also 4.3%. As only 1 person was positive in 5 separate families intrafamilial transmission between nonexposed humans seems unlikely. That demonstration of MRSA CC398 in humans living on farms without exposure to colonized pigs is rather due to acquisition from exposed, colonized family members and not due to dissemination in the community at country side is supported by the results from studying MRSA colonization of pupils in an agricultural area (see below). The age distribution of MRSA positive humans exposed to pigs corresponded to the active working age of farmers: 52 from 59 between 30 and 60, 19 from 27<30, 10 from 11>60 years. For MRSA positive humans among the family members this distribution was 2 from 72<30 years, 1 from 26 between 30 and 60 years, and 1>60 years.

**Table 1:** Nasal carriage of MRSA CC398 among families living on pig farms.

Category	Subcategory	Result
Carriage of MRSA CC398	Total number of families:	57
	Number of families on farms with MRSA positive pigs	47
	Number of humans exposed to MRSA positive pigs	113
	Nasal carriers among persons exposed to MRSA positive pigs	97 (86%)
	Number of non exposed family members living in contact to exposed persons carrying MRSA	116
	MRSA carriers among non exposed contact persons	5 (4.3%)
Transfer events	Number of families with transfer events	5
	Number of transfer events to non exposed humans	5
	Frequency of transfer events in relation to all non exposed humans	4.3%

### Carriage of MRSA among veterinarians attending pigs and among their family members

These results are shown in table 2. Among 49 veterinarians who are attending the 47 pig farms under study 22 revealed as nasal carriers of MRSA CC398 (45%). Eighteen among them associated with 15 families agreed to investigate their family members for MRSA nasal carriage. None of these families was living on a farm or raising livestock. Altogether 4 from 44 nonexposed contact persons, each in a separate family, revealed as positive for MRSA CC398 (9%) exhibiting the same *spa*-types and antibiotic resistance phenotypes as the MRSA isolates from their exposed family members. As no transfer among nonexposed family members was observed, the frequency of transfer from exposed to nonexposed family members was 9%.

**Table 2:** Nasal carriage of MRSA CC398 among veterinarians attending pigs and among their family members.

Category	Subcategory	Result
Carriage of MRSA CC398	Total number of veterinarians:	49
	Nasal carriers of MRSA	22 (45%)
	Number of families investigated for MRSA carriage	15
	Number of family members exposed to pigs and carrying MRSA	18
	Number of family members living in contact to exposed persons carrying MRSA	44
	Number of MRSA carriers among non exposed contact persons	4 (9%)
Transfer events	Number of families with transfer events	4
	Number of transfer events to non exposed humans	4
	Frequency of transfer events in relation to all non exposed humans	9%

### Influence of previous antibiotic treatment on carriage of MRSA CC398 in families living on farms

As shown in table 3 antibiotic treatment was reported by 29 persons from 102 who were colonized with MRSA, and by 61 among the 127 non colonized ones.

**Table 3:** Antibiotic treatment previous to sampling of pig farmers and of their non exposed familial contact persons.

Category	Subcategory	MRSA positive	MRSA negative
humans exposed (n = 113)	antibiotic treatment	28	4
	no antibiotic treatment	69	12
humans non exposed (n = 116)	antibiotic treatment	1	57
	no antibiotic treatment	4	54
**Sum:**		**102**	**127**

In univariate analysis both exposition to colonized pigs as well as antibiotic treatment revealed as risk factors for MRSA colonization of humans. In multivariate analysis only exposure to colonized pigs remains as a significant risk factor (Table 4).

**Table 4:** Statistical analysis.

Category	Subcategory	Result
variable exposition	univariate analysis	168,2 (54,4 – 520,3) p<0,001, RR 95% CI
	multivariante analysis	158,2 (54,4 – 520,3) p>0,01, 95% CI
variable antibiotic treatment	multivariante analysis	0,457 (0,264 – 0,793) p 0,05, RR 95% CI

### Transmission of methicillin susceptible Staphylococcus aureus (MSSA) among family members not exposed to pigs

Among the 57 families investigated nasal colonization with MSSA was found in 25 of them. Families with MSSA carriers consisted altogether of 124 persons. Colonization with MSSA was detected in 45 of them. Co-colonization with MRSA was not detected. Typing of the MSSA isolates indicated 14 transfer events in 10 families with 1 transfer event in each one of 8 of them, and two transfer events in each of 3 other families. The frequency of transfer events was 11%. These data are shown in table 5. The following *spa*-types were observed (number of isolates/family(ies): t002: 1/1; t005: 3/2, t008: 3/1, t011: 1/1; t012: 1/1, t015: 2/1, t021: 1/1, t034: 5/4, t040: 1/1, t056: 3/1, t089: 1/1, t091: 2/2, t127: 1/1, t166: 2/2, t493: 3/1, t778: 1/1, t779: 1/1, t859: 1/1, t1430: 2/1, t2582: 1/12 t1731: 3/1, t2582: 1/1, t2828: 1/1, t2922: 1/1, t3374: 2/1, t3828: 1/1, t4107: 1/1, t4753: 1/1.

**Table 5:** Nasal carriage of methicillin susceptible *S. aureus* (MSSA) among families living on pig farms.

Category	Subcategory	Result
Carriage of MRSA CC398	Total number of families:	57
	Number of families with MSSA carriers	25
	Total number of humans in families with MSSA carriers	118
	Number of MSSA carriers	45 (38%)
Transfer events	Number of families with transfer events	10
	Number of transfer events	12
	Frequency of transfer events in relation to all family members	10%

Besides *spa*-type t005 which was exhibited by isolates from 3 members of 2 from 27 families the other *spa*-types were detected only one time in each of the 27 families and are rarely represented by *S. aureus* in general (database www.ridom.de). Therefore transmission between different family members seems likely.

### Dissemination of MRSA CC398 beyond pig farms in the community

For addressing this question nasal swabs were taken from pupils at the average age from 10 to 16 years living in villages in the high density pig farming area in North-Rhine Westphalia and attending a secondary school in small town R. Twenty-seven from all of the 47 the farms studied for MRSA are located in this area. From 462 pupils only 3 were found to carry MRSA CC398. These pupils were 3 from 40 ones living on pig farms. The other 422 pupils investigated belonged to families not living in the farm environment (association of MRSA colonization with living on pig farms, p = 0,001 according to Fisher's exact test).

### Results from *spa*-typing and from antibiotic susceptibility testing of MRSA

For all MRSA isolates typed (n = 921) *spa*-types could be assigned to clonal complex CC398, the following types were observed: t011 (56%), t034 (33%), t2974 (4.4%), t108 (2.2%), t1197 (1.8%), t571 (0.7%), t1451 (0.7%).

All of the MRSA isolates tested (n = 921) exhibited resistance to oxytetracycline. Multiresistance phenotypes are shown in table 6. There was no association between particular spa-types and antibiotic resistance patterns. All of the isolates were susceptible to daptomycin, fosfomycin, fusidic acid, linezolid, rifampicin, tigecyclin, teicoplanin, vancomycin, linezolid and to mupirocin.

**Table 6:** Resistance phenotypes of MRSA CC398 originating from pigs and from humans at 47 farms investigated from September 2007 – December 2008 in Germany.

Resistance phenotype	Number of isolates
pen, oxa, ote, ery, cli	276 30%
pen, oxa, ote	163 18%
pen, oxa, ote, sxt	110 12%
pen, oxa, ote, sxt, gen	110 12%
pen, oxa, ote, ery, cli, sxt	105 11%
pen, oxa, ote, sxt, gen, ery, cli	96 10.4%
pen, oxa, ote, ery, cli, gen	15 1.6%
pen, oxa, ote, cip	14 1.5%
pen, oxa, ote, ery, cli, cip	8 0.9%
pen, oxa, ote, gen	7 0.8%
pen, oxa, ote, cip, mfl	7 0.8%
pen, oxa, ote, ery, cli, sxt, cip, mfl	7 0.8%
pen, oxa, ote, cli, cip	3 0.3%
	**921**

CIP = ciprofloxacin, CLI = clindamycin, ERY = erythromycin, GEN = gentamicin,

MFL = moxifloxacin, OTE = oxytetracycline, OXA = oxacillin, SXT = cotrimoxazole.

## Discussion

Nasal carriage of MRSA CC398 by humans with professional contacts to pigs colonized with MRSA of this clonal complex has been reported for veterinarians [18], [19] in Europe and for swine farmers in the USA [11]. The colonization frequency of farmers observed by us was nearly 3 times as high as reported from a recent study in the Netherlands [23]. We cannot exclude methodological reasons as the Dutch investigators used selective enrichment broth containing oxacillin (low *in vitro* expression of heteroresistance in case of testing oxacillin in comparison to cefoxitin [30] which is used in selective chromogenic agar). Also differences in the protective effect of natural colonization with methicillin susceptible *S. aureus* (MSSA) and other competing microflora can not be excluded.

As shown by previously performed analysis in the Netherlands [23] for nasal colonization and for cases of infections in Denmark [22] exposure to MRSA colonized pigs is an independent risk factor for acquisition of MRSA CC398 by humans. According to our results the risk to get colonized is 168 times higher for humans on farms with regular daily exposure then for nonexposed humans living on farms. Univariate regression analysis shows a slightly higher risk for getting colonized with MRSA CC398 due to antibiotic treatment. As, however, revealed by multivariate analysis, antibiotic treatment has no influence on MRSA colonization in case of humans exposed to MRSA colonized pigs. This has also been observed by other investigators [11].

Colonization with MRSA CC398 of nonexposed humans living on farms is low (4.3%), this confirms results from the previous study performed in the Netherlands (2% [23]). The results from typing of the isolates suggest that MRSA CC398 has the capacity for transmission among humans living in close contact. Family clusters have also reported from a study in Denmark (22). One could argue that MRSA CC398 could have been acquired by not directly exposed humans via dust blown out of the pig stables, by touching devices taken out, or by contact to items taken home from the stable. Nasal carriage of MRSA CC398 by veterinarians has also been reported by previous studies on congress participants with higher colonization rates for those who attend pigs and calves [19]. We observed a lower colonization frequency of veterinarians in comparison to farmers, this might be due to different degrees of exposition.

Of particular interest is the comparison to the frequency of transfer events of MSSA among nonexposed humans living on pig farms which was twice as high as for MRSA CC398.

Only in case of isolates exhibiting *spa*-type t005 one could argue that this spa type is frequently represented among *Staphylococcus aureus* from nasal carriage (∼10% among in a study performed in Germany) that members of the same family have acquired their colonizers independently. For the other isolates exhibiting *spa*-types which are infrequent in nasal carriers [31] transmission between family members is likely. Household transmission of *Staphylococcus aureus* seems not to be uncommon [32]. MSSA (PCR for *mec*A and for *ccr*C was negative) exhibiting *spa*-types 11 and 34 which attribute them to CC398 were found in 5 nonexposed humans belonging to 4 different families suggesting 1 transfer event. It remains unclear whether these isolates represent susceptible ancestors of MRSA CC398 or derivatives having lost the SCC*mec* element.

Human to human transmission of MRSA CC398 seems also to be less frequent than that reported for hospital associated MRSA in families of health care workers attending dialysis patients [33]. There is obviously also less frequent transmission of MRSA CC398 among patients in the hospital setting [34]. As suggested by the results from studying colonization of pupils in an agricultural area, dissemination of MRSA CC398 among humans outside farms is apparently limited.

Meanwhile several authors reported that MRSA CC398 is able to cause infections in humans [9], [21], [22], [35]. Severe skin and soft tissue infections (SSTI) caused by MRSA CC398 are comparable to those caused by caMRSA with respect to surgical intervention and even admission to a hospital [22], [36]. So far MRSA CC398 seems still to be infrequent among MRSA isolates from SSTI in the community. Only 3 from 83 of them sent to our laboratory for typing in 2008 were assigned to CC398 (for the others: 38 to CC8, 30 to CC80 and 10 to other clonal complexes, unpublished results from the author's laboratory).

Several aspects need further studies. So far there are only few data on the stability of nasal colonization with MRSA CC398 in the absence of exposure to pigs. Future surveillance has to address the question of progressing adaptation to humans with respect to dissemination beyond farms and to introduction and spread in the hospital as it has already been observed in the Netherlands [36].

According to data exciting so far measures for prevention of infections in humans by MRSA CC398 should focus on humans with direct exposure to livestock. Emergence of skin and soft tissue infections need the same attention as infections with caMRSA with respect to surgical interventions, antibiotic treatment if necessary and measures of personal hygiene [37]. Regimens for screening ad admission to hospitals should include farmers raising productive livestock and veterinarians with subsequent precaution measures as it has already been recommended in the Netherlands [38] and in Germany [39]. In case of elective treatment in hospitals a check on nasal colonization ahead of admission with subsequent sanitation in case of a positive result is highly advisable.

Farmers raising livestock should be informed that when admitted for emergency treatment they should announce their probable MRSA colonization for reason of targeted calculated antibiotic treatment in case of an infection. Although already resistant to different classes of antibiotics MRSA CC398 exhibit a susceptibility pattern that still leaves a number of therapeutic options.
